# Use of surrogate species to cost‐effectively prioritize conservation actions

**DOI:** 10.1111/cobi.13430

**Published:** 2019-12-26

**Authors:** Michelle Ward, Jonathan R. Rhodes, James E.M. Watson, James Lefevre, Scott Atkinson, Hugh P. Possingham

**Affiliations:** ^1^ Centre for Biodiversity and Conservation Science, School of Biological Sciences The University of Queensland Brisbane QLD 4072 Australia; ^2^ School of Earth and Environmental Sciences The University of Queensland Brisbane QLD 4072 Australia; ^3^ Wildlife Conservation Society Global Conservation Program 2300 Southern Boulevard, Bronx New York NY 10460 U.S.A.; ^4^ Institute of Molecular Bioscience The University of Queensland Brisbane QLD 4072 Australia; ^5^ United Nations Development Programme 1 United Nations Plaza New York NY 10017 U.S.A.; ^6^ The Nature Conservancy Minneapolis MN 55415 U.S.A.

**Keywords:** conservation planning, conservation shortcuts, cost‐effective analysis, prioritization, surrogacy, threat management, threatened species, umbrella species, análisis rentable, atajos de conservación, especies amenazadas, especies paraguas, manejo de amenazas, planeación de la conservación, priorización, sustitución, 保护规划, 保护的捷径, 成本效益分析, 优先保护, 替代物种, 威胁管理, 受胁迫物种, 伞护种

## Abstract

Conservation efforts often focus on umbrella species whose distributions overlap with many other flora and fauna. However, because biodiversity is affected by different threats that are spatially variable, focusing only on the geographic range overlap of species may not be sufficient in allocating the necessary actions needed to efficiently abate threats. We developed a problem‐based method for prioritizing conservation actions for umbrella species that maximizes the total number of flora and fauna benefiting from management while considering threats, actions, and costs. We tested our new method by assessing the performance of the Australian federal government's umbrella prioritization list, which identifies 73 umbrella species as priorities for conservation attention. Our results show that the federal government priority list benefits only 6% of all Australia's threatened terrestrial species. This could be increased to benefit nearly half (or 46%) of all threatened terrestrial species for the same budget of AU$550 million/year if more suitable umbrella species were chosen. This results in a 7‐fold increase in management efficiency. We believe nations around the world can markedly improve the selection of prioritized umbrella species for conservation action with this transparent, quantitative, and objective prioritization approach.

## Abstract 2

*Article impact statement*: Considering spatially overlapping species, threats, actions, and costs in conservation prioritization yields 7‐fold increase in efficiency.

## Abstract (es)

Uso de Especies Sustitutas para Priorizar las Acciones de Conservación de Manera Rentable

**Resumen**

Los esfuerzos de conservación comúnmente se enfocan en las especies paraguas cuyas distribuciones se traslapan con la de muchos otros componentes de la flora y fauna. Sin embargo, como la biodiversidad se ve afectada por diferentes amenazas que presentan una variabilidad espacial, enfocarse solamente en el traslape de la extensión geográfica de las especies puede no ser suficiente para la asignación de las acciones necesarias para abatir eficientemente a las amenazas. Desarrollamos un método basado en problemas para la priorización de las acciones de conservación para las especies paraguas, el cual maximiza el total de flora y fauna beneficiado con el manejo a la vez que considera amenazas, acciones y costos. Probamos nuestro método al valorar el desempeño de la lista de priorización de especies paraguas del gobierno federal de Australia, la cual identifica a 73 especies paraguas como prioritarias para la atención de la conservación. Nuestros resultados muestran que la lista de prioridades del gobierno federal beneficia sólo al 6% de las especies terrestres amenazadas en Australia. Esto podría incrementarse para beneficiar a casi la mitad (o el 46%) de las especies terrestres amenazadas con el mismo presupuesto de AU$550 millones al año si se eligieran a especies paraguas más adecuadas. Esto resulta en un incremento siete veces mayor en la eficiencia del manejo. Creemos que en todo el mundo los países pueden mejorar de una manera muy marcada la selección de las especies paraguas para las acciones de conservación con una estrategia de priorización transparente, cuantitativa y objetiva.

## 摘要

保护工作常常聚焦于分布区与许多其他动植物重叠的伞护种。然而, 由于生物多样性受到各种空间上可变威胁的影响, 因此, 仅关注物种地理范围的重叠不足以合理分配有效减缓威胁所需的必要行动。我们开发了一种具体问题具体分析的方法, 来确定在考虑威胁、行动和成本的情况下, 可以使最多的动植物从管理中获益的伞护种优先保护行动。本研究通过评估澳大利亚联邦政府包含 73 个优先保护伞护种的保护名录, 对这一新方法进行了测试。结果表明, 联邦政府的优先保护名录只惠及 6% 的澳大利亚濒危陆生物种。如果选择更合适的伞护种, 同样利用每年 5.5 亿澳元的预算, 可以将这一比例提高到接近一半 (46%) 的濒危陆生物种, 这也意味着管理效率可以提升 7 倍。我们相信, 利用这个透明、定量和客观的优先保护排序方式, 世界各国都可以显著改善对优先保护的伞护种的选择。**【翻译: 胡怡思; 审校: 聂永刚】**

## Introduction

Species extinction is one of the most significant environmental challenges humanity faces (Ceballos et al. 2017); rates are up to 1000 times higher than what is considered natural (De Vos et al. 2015). In response to this challenge, most countries have ratified the Convention on Biological Diversity (CBD) and the 2020 Strategic Plan for Biodiversity (United Nations Convention on Biological Diversity 2010). These plans commit all signatories to prevent extinction of known threatened species and improve their conservation status by 2020. A critical factor of success in achieving these plans is financing, yet globally there is inadequate investment in conservation (Waldron et al. 2017). With an increasing species extinction crisis (IPBES 2018), a looming CBD deadline (United Nations Convention on Biological Diveristy 2010), and limited conservation funding globally (Waldron et al. 2017), better methods to prioritize investment of resources in species recovery are needed.

Many governments, conservation agencies, and scientists use umbrella prioritization approaches to achieve conservation goals efficiently (e.g., Rowland et al. 2006; Rodrigues & Brooks 2007; Caro & Girling 2010). This approach prioritizes flora and fauna based on their large habitat requirements (Wilcox 1984), thereby minimizing the need to target all species within a landscape by managing just 1 that encapsulates many others with overlapping habitat requirements (Noss 1990; Launer & Murphy 1994). This approach has been used in a variety of efforts, including the delineation of Katavi National Park boundary in Tanzania (Caro 2003), the Conservation Threatened Species Program in New Zealand (New Zealand Government 2015), and Australia's Threatened Species Strategy (Australian Government 2015). One example of an umbrella species is China's giant panda (*Ailuropoda melanoleuca*). China prioritizes an extensive nature reserve network for this single species that encompasses the ranges of all but 1 vertebrate endemic to central China (Li & Pimm 2016). Although this approach is often simple to understand by policy makers (and as such often quick to implement), it is primarily based on the distribution of species and often does not consider threats, actions to mitigate threats, or costs of actions. Threats are species specific. For example, an action for an umbrella species that aims to remove a specific threat does not necessarily help other species merely because the species have overlapping ranges. Although there has been extensive research on umbrella species that defines the method (Caro & Girling 2010), validates umbrella species as an efficient conservation tool (e.g., Nekaris et al. 2015; Maslo et al. 2016; Kalinkat et al. 2017), analyzes the effectiveness of specific umbrella species (e.g., Bifolchi & Lodé 2005; Rowland et al. 2006; Li & Pimm 2016), and alternative threat‐based approaches (e.g., Lambeck 1997), it is still unknown how efficiency changes in an umbrella species approach when threats, actions, and costs are considered in a systematic assessment.

We used decision‐analysis methods for targeting investment in management of threatened species (e.g., Joseph et al. 2009; Carwardine et al. 2012; Chadés et al. 2015) to quantify the efficiency of prioritizing species’ management while considering geographic range and threat overlaps. We calculated efficiency based on the number of species that could be managed under a strict budget and prioritized species management based on those that are the most umbrella effective (i.e., species that can simultaneously benefit many other flora and fauna from the management of itself). We tested this method on the Australian federal government's recent plan for prioritizing 73 threatened species, which is part of a wider effort to protect and recover all plants and animals at risk of extinction. We believe that our problem‐based prioritization approach to choose umbrella species is applicable to other regions that contain many imperiled species.

Australia has approximately 1828 threatened species and one of the highest extinction rates on Earth (Woinarski et al. 2011). The prioritized species were chosen by the federal government because they were thought to be important umbrella species (Australian Government 2015), but it is unknown how effectively they represent all threatened flora and fauna.

## Methods

### Management of Threatened Species in Australia

Australia is a megadiverse country due to its high level of endemism (Department of Environment and Energy 2018). Approximately 85% of its flowering plants, 84% of its mammals, 45% of its birds, and 89% of its reptiles are endemics (Department of Environment and Energy 2018). However, Australia is experiencing a biodiversity crisis; approximately 1828 species (of which 1777 are terrestrial species) are listed under the federal Environment Protection and Biodiversity Conservation (EPBC) Act 1999 (herein, referred to as threatened species), of which 91 have gone extinct since European arrival (Australian Government 2018). The Australian Government has recently mobilized 2 funding programs for threatened species. These include $50 million for the Threatened Species Prospectus and $5million for a Recovery Fund program, of which 19 projects have approval for $3 million (Department of Environment and Energy 2018). (All monetary units are in Australian dollars unless otherwise noted.) Funding for 73 of the 1828 species is prioritized.

### Threatened Terrestrial Species Data

To solve the problem of prioritizing the best suite of umbrella species, we used Australian spatial distribution data that have been applied in many international studies (Guisan et al. 2013; Auerbach et al. 2015; Polak et al. 2016). We created a binary matrix of all 1777 threatened terrestrial species in the data set to identify species‐specific threats taken from the Australian government's online Species Profile and Threats Database (Australian Government 2016).

### Objective Function

Using an iterative greedy algorithm calculated in R 3.4.4, we produced a list of species and associated actions in the order in which they are most cost‐effective. This list of species maximized the total number of flora and fauna that could benefit from implementation of certain management actions while remaining within a strict budget. Our model accounted for how many other species benefit when umbrella species are managed, and we assumed that all threats to the umbrella species are managed across its entire range. Though this greedy algorithm was not perfectly optimized, it is easy to implement and replicate in any nation or landscape. The cost‐effective formula of managing all threats to umbrella species *i* is

(1) $$\;\;E_{i}=\frac{B_{i}\;}{C_{i}},$$

where $B_{i}$ is the benefit to the chosen species and all other species (Eq. 2) and *C_i_* is the cost of managing all threats to recover species *i* (Eq. 3).

We prioritized management in order of cost‐effectiveness; thus, not all species were identified as umbrella species. For example, some species may only benefit themselves and have 1 inexpensive threat and thus rank high in cost‐effectiveness. Therefore, we categorized species as umbrella species, benefiting species, and additional species. Umbrella species were those that have geographic and threat overlap with other flora and fauna, and conservation actions can be implemented cost‐effectively. Benefitting species are defined as those that can benefit simultaneously from actions implemented for an umbrella species. Additional species are those that are added to the list in order of cost‐effectiveness until the budget is met, even though they do not contribute to the conservation of any other species.

### Benefit Function

**Figure 1:**
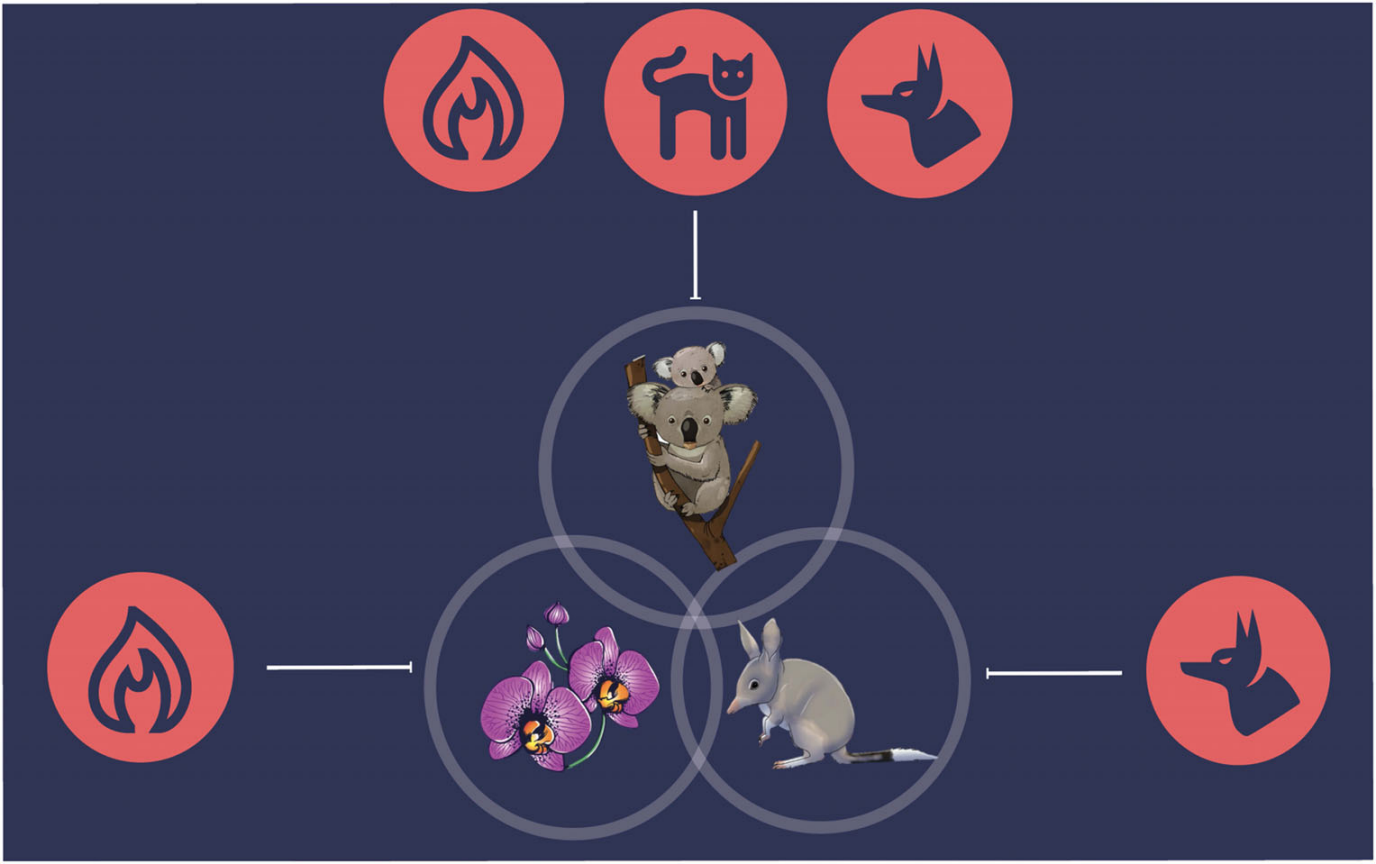
The baseline scenario of accumulated benefit of a surrogate species based on proportion of overlapping range and matching threats. In this example, koala (Phascolarctos cinereus) receive a benefit of 0.15 (i.e., 15% of its distribution) when foxes (Vulpes vulpes) are managed to protect greater bilby (Macrotis lagotis) and a benefit of 0.15 (i.e., 15% of its distribution) when fire is managed to protect orchids (Orchis spp.). Management of greater bilby and orchid would not be managed further than 100% of distribution, and koala would receive a benefit of 1.30, including the benefit to itself.

We calculated the benefit function in 3 different ways: baseline scenario, optimistic scenario, and pessimistic scenario. We report only the baseline scenario results in the text, but methods and results for all 3 scenarios are given in the Supporting Information. In each case, the benefit of any action is accrued to all species affected by a particular threat and that had overlapping ranges. A species could benefit from a threat‐specific action only once, so there was no double counting of benefit. For example, if species *i* is a potential umbrella species and its distribution overlaps *p* other species, then the overlapping area is counted in *p* overlap terms. Once *i* species is managed, it cannot be managed again. As such, there is no duplication, just a representation of the benefit to each of the *p‐*1 species (or other species in the area). Under the baseline scenario, the benefit function is

(2) $$B_{i}\sum_{j=1}^{n}\left(\frac{O_{\mathrm{ij}}W_{i}}{\sum_{k=1}^{m}T_{jk}}\sum_{k=1}^{m}T_{ik}T_{jk}\right),$$

where *B_i_* is the benefit of managing all threats for species *i* *; n* is the number of species; *O_ij_* is the percentage of overlap between species *i* and *j; W_i_* is the weight, which was set to 1 for all species, but can be varied based on factors, such as taxonomic uniqueness; *m* is the number of threats; $T_{ik}\;$is a binary variable indicating whether threat *k* is a threat for species *i* (similarly for *T_jk_*); and *k* is a specific threat. For the baseline scenario, we assumed that if we managed a threat over only a fraction of a species’ range, then it would receive a proportional benefit (Fig. 1). This is a reasonable assumption in that our costed actions remove a threat; therefore, the actions benefit all species affected by that specific threat where the ranges overlap (Auerbach et al. 2015; Bennett et al. 2015). Ideally, we would have quantitative data on how partial management of each action benefits each species, including time frames. However, until that information is available for all species, a linear benefit assumption seems reasonable (Woinarski et al. 2007; Groom 2010; Carwardine et al. 2012; Auerbach 2015).

### Cost Constraints

As threats are managed for increasingly larger areas, the per unit area cost usually declines (Armsworth et al. 2018). Hence, we used a diminishing returns function, where the marginal cost decreases as the area of management increases. We used a power‐law function to represent this relationship, whereby the total cost of an action ($C_{i}$) is a function of the area managed, $R_{i}$ for species *i*:

(3) $$\;C_{i}=\;q{R_{i}}^{z},$$

where *q* is a parameter fitted using data that transforms area into cost and $R_{i}$ is the range over which the threat is managed for species *i*, in this case the size of the range of the target species. To find *q*, we conducted a literature review in July 2019. We collated articles and reports by searching Scopus for the terms “*biodiversity* and *manage* ^*^ and *Australia* ^*^ and *cost”* for all years to July 2018. To be included, the article or report must have identified costs of actions to mitigate threats, and costs had to be in Australian dollars. Of the 310 articles found, 15 papers were retained. These amounts were recalculated to 2018 costs based on the Australian consumer price index (www.rba.gov.au/inflation/measures-cpi.html#quarterly; Iacona et al. 2017) and rescaled to the median cost per square kilometer per year (all cost estimates are given in the Supporting Information). If a threat did not have an action to either manage or eliminate, the benefit was 0 and assumed to be not managed. Typical *z* values are from 0.15 to 0.40, depending on habitat type and distribution of species (Murdoch et al. 2007). Because we did not have an economic basis for parameterizing the value of *z*, we evaluated a range of sequential values ranging from 0.15 to 0.40 to reflect the uncertain relationship between cost and area (Murdoch et al. 2007; Wilson et al. 2007). The *z* values we used were 0.20, 0.30, and 0.40, all of which were separately explored under all 3 scenarios (only 0.30 is reported). We also explored the sensitivity of these *z* values on the overall priority species lists.

### Calculation of Assumed Budget

To identify the difference in effectiveness between our optimized priority list and the federal government's priority list, we first calculated the cost of actions to manage all threats affecting the government's 73 priority species with our baseline scenario benefit function. The total cost of this management provided an assumed budget, which we used to constrain our new problem‐based approach. We then calculated the number of species that could be managed as umbrella species, benefitting species, and additional species under the above‐mentioned assumed budget.

### Prioritization Solution

Our objective was to maximize the total number of flora and fauna that could benefit from threat management by implementing specific conservation actions. We formulated this as a maximum gains decision problem by representing the set of species to specifically manage with the vector **x**, where *x_i_* = 1 if species *i* is targeted for management as an umbrella species and 0 otherwise. We then sought to determine

(4) $$\max\;\sum_{i=1}^{n}B_{i}\left(x\right)\;,$$

*s.t. ∑* $x_{i}C_{i}\leq \;$budget,

where $B_{i}$ (**x**) is the benefit for managing species *i* (see Eq. 2) as a function of the vector **x**, which provides a prioritized list of umbrella species we are acting on, and $C_{i}$ is the cost of managing all threats to species *i* across its entire range (Eq. 3). This is subject to (s.t.) a budget. We then found an approximate solution to this formula by using a greedy algorithm. This greedy algorithm aimed to select the most beneficial threatened species (species whose management benefits many other threatened species) that cost the least to manage (Possingham et al. 2000; Polasky et al. 2001). This algorithm identified threatened species with the highest cost‐effectiveness (*E_i_* = *B_i_/C_i_*) when all threats are managed. The model then sequentially searched for the next most cost‐effective threatened species that increases the net benefit (Eq. 2). The process stopped when the desired budget was reached. The final outcome was a list of species and actions that approximated the strategy that maximizes the total number of flora and fauna benefiting from management for a given budget.

## Results

### Cost‐Effective Government Priority Species

**Figure 2:**
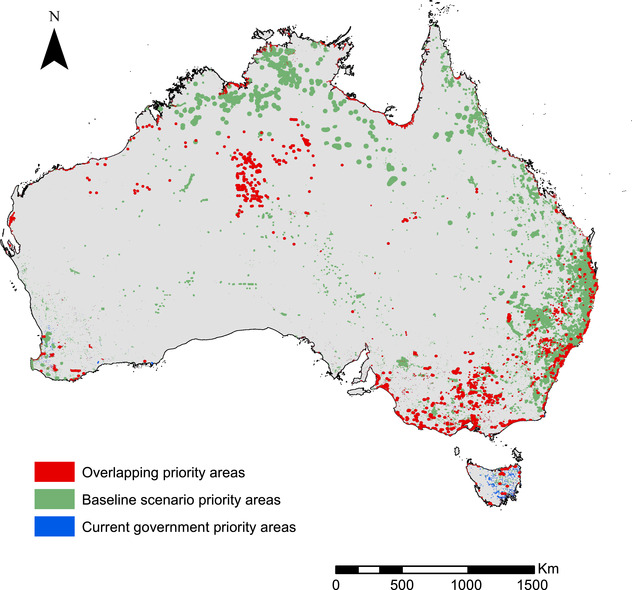
Spatial distribution of priority areas to manage in Australia based on the federal government's priority species distributions; our optimized list of priority umbrella species distributions; and species distributions that overlap geographically with government priority areas and the optimized priority areas. (Map developed in ArcGIS 10.4.)

We found that management of all threats throughout the distribution of the government's 73 priority species would cost approximately $550 million/year. The net benefit, defined as the total number of threatened flora and fauna expected to benefit under management, was 103 species (6% of all Australia's terrestrial threatened species). Management of 43 of the 73 government priority species was cost‐effective, and these species were retained on the optimized list. These included 12 birds, 20 plants, and 11 mammals (distributions in Fig. 2).

### Optimizing Management

**Figure 3:**
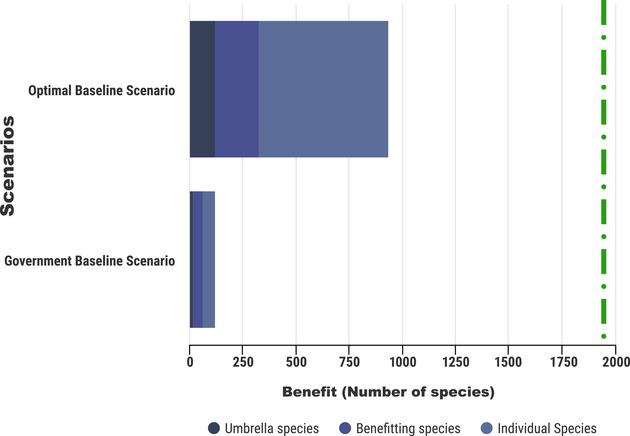
Number of umbrella species (species that have geographic and threat overlap with other flora and fauna and conservation actions can be implemented cost‐effectively), benefitting species (i.e., species that can benefit simultaneously from actions implemented for an umbrella species), and additional species (i.e., species added to the list in order of cost‐effectiveness until the budget is met, even though they do not contribute to the conservation of any other species). Numbers are based on the baseline scenario, median costs of managing threats, and diminishing returns *z* value of 0.30 (green line, total number of threatened species in Australia).

Using our iterative optimization model, the number of species that benefited increased to 816 (46% of all threatened terrestrial flora and fauna in Australia) under the assumed government budget (Fig. 3). Of these 816 species, 120 were classified as umbrella species, 208 were benefitting species, and 608 were additional species. This is an increase of 690% in species benefitting from management compared with the government priority list, all while remaining within the assumed budget of $550 million/year.

### Cost‐Effective Umbrella Species

**Figure 4:**
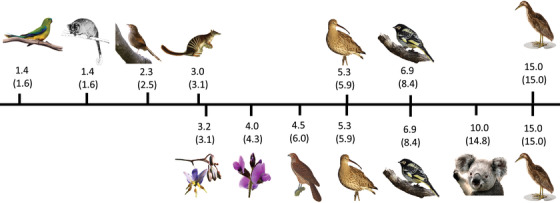
Number of species benefitting from management when the 7 most umbrella‐efficient species are managed from the Australian government's priority list (top) and from the optimized list of priority species (bottom) (numbers in parentheses, species that could be managed without considering the umbrella benefits of any other species; benefit line is not to scale).

The species providing the most benefit to other terrestrial threatened fauna and flora was Australasian Bittern (*Botaurus poiciloptilus*). This wide‐ranging bird is endangered on the government's threatened species list. The population is decreasing, yet when managed for all 7 threats (fire, habitat loss, pollution, feral cats, grazing livestock, and high salinity) its management benefited 15 other threatened species that are also negatively affected by these threats (Fig. 4). Australasian Bittern required approximately $2.3 million/year to manage all threats across its entire range. When Koala (*Phascolarctos cinereus*) were managed for all the threats it faces, it benefited 10 additional threatened species at a cost of $4.6 million/year. Management of Regent Honeyeater (*Anthochaera phrygia*) and Far Eastern Curlew (*Numenius madagascariensis*) threats benefited an additional 7 and 5 species, respectively. Management of all threats affecting Regent Honeyeater costs approximately $2.6 million/year, and management of Far Eastern Curlew costs approximately $2.3 million/year. Management of Red Goshawk (*Erythrotriorchis radiates*) benefited 5 additional threatened species for $2.4 million/year, and purple clover (*Glycine latrobeana*) management benefited 4 additional threatened species and costs only $940,000/year. Matted flax‐lily (*Dianella amoena*) was also a highly umbrella‐effective species. Its management benefited 3 threatened flora and fauna and costing approximately $690,000/year. Despite koala, Red Goshawk, matted flax‐lily, and purple clover being highly beneficial species, they do not appear on the government priority list. These species were the 7 most effective umbrella species when assessed based on complimentary benefit of actions, but when evaluated based on their individual management impact (i.e., prior umbrella benefits removed), the number of benefitting species changed (Fig. 4). This indicated that some of the most effective umbrella species shared geographic ranges and threats with each other.

### Cost Function Sensitivity Analyses

After exploring the sensitivity of the 3 different cost function relationships, with *z* = 0.2, 0.3, 0.4 (Eq. 3), for the overall priority species lists, we found negligible changes in the membership of the final priority species lists. Under the baseline scenario (which assumes that benefit is proportional to the overlap), species listed as priorities remained consistent 97% of the time. This indicates that although there was an uncertain relationship between cost and area, it had little effect on the overall choice of priority umbrella species.

## Discussion

There is an emerging, critical role for prioritization approaches to be efficient and achieve conservation goals that maximize the number of species benefitting from management (Nicholson & Possingham 2006; Rodrigues & Brooks 2007). In this case study, our new method delivered a 7‐fold increase in management efficiency while remaining under the assumed budget. We found that an umbrella species approach that considers geographic and threat overlap, species‐specific threats, actions needed to mitigate threats, and costs of actions within a properly formulated decision problem can objectively and efficiently prioritize actions. This efficiency occurred because we removed replicated management and cost redundancy, which are goals of an umbrella‐species approach (Tulloch et al. 2013; Chadés et al. 2015).

Using our optimization approach to identify umbrella flora and fauna, we increased government management benefits and stayed within the assumed budget. This budget of $550 million/year seems reasonable, considering other research indicates that the budget should be $842 million–2.5 billion/year to recover all Australian species threatened with extinction (Wintle et al. 2019). Although we used the best available information on costs of possible actions in Australia, detailed costing or feasibility analyses for each action is still required within a local context.

Some argue that incorporating the cost of management into decision making may lead to high conservation value areas being overlooked due to cheaper options being prioritized (Naidoo et al. 2006; Armsworth et al. 2018). In our study, the highest priority species were a mix of classic wide‐ranging umbrella species (e.g., koala with a distribution of 248,700 km^2^) whose cost‐effective management benefited many other species and species with narrow distributions (e.g., *D. amoena* with a distribution of 544 km^2^) that are cheap to manage and overlap with a few other species with narrow distributions. We showed that by combining both transparent expenditure and expected benefits to species, decision makers can make rational, efficient, and informed prioritization choices that maximize conservation outcomes (Iacona et al. 2018).

Although prioritization and efficiency are not needed if all threats and all biodiversity can be managed, the majority of countries do not have the resources (Scheele et al. 2018) or lack political will (Macintosh & Wilkinson 2005; Kati et al. 2015) to adequately recover all species. For example, in the United States, which has the largest nominal gross domestic product in the world (World Bank 2018), 1662 plants and animals are threatened with extinction. To date, the U.S. government has spent approximately US1.45 billion/year (USFWS 2016) and has had 39 species recover, down‐listed from endangered to threatened, or removed entirely from the threatened species list (USFWS 2016; USFWS 2019; Wintle et al. 2019). This results in a total recovery of 2.4% of all threatened species. Australia has had substantially less success, with only a few species recover as a result of management (Scheele et al. 2018). Despite its great economic wealth, relatively good governance, and world‐class scientific expertise (Mcdonald et al. 2015), Australia does not adequately invest in recovery plans (Walsh et al. 2013), management, or monitoring of its most imperiled species (Wintle et al. 2019). Therefore, prioritization methods that maximize efficiency are necessary because there is generally limited availability or allocation of resources toward conservation (Joseph et al. 2009).

Using our problem‐based model, decision makers can explore different conservation objectives through the use of weights within the benefit function. We weighted each species to 1, thereby treating all species equally. This equality allowed us to meet our primary objective of maximizing the number of species managed within a fixed budget. Some decision makers, however, may wish to bias results toward certain species that have the best chance of recovery, are taxonomically distinctive, culturally significant, or have been identified as flagship species (Joseph et al. 2009; Bennett et al. 2014). This additional weighting can be altered easily and in a transparent way within our model, and trade‐offs of such decisions can be explored. For example, trade‐offs may exist between maximizing the greatest number of species managed versus a higher number of species that are endemic to a nation (Joseph et al. 2009).

Decision makers may also wish to explore different ways to calculate the benefit function. We investigated 3 scenarios that considered how species could benefit from different levels of management. These included the baseline scenario, which assumes the benefit is proportional to the overlap, an optimistic scenario, whereby only 1 threat had to be managed to gain a benefit for a species, and a pessimistic scenario, under which we assumed that all threats must be managed throughout their entire geographic range (see Supporting Information for more detail). Our baseline scenario provided the most realistic list of priority umbrella species. For example, it is reasonable to assume that management of Australasian Bittern, a wide‐ranging partially nocturnal heron affected by 7 different threats, can benefit 15 other species within its range. Yet it is unrealistic to believe that the management of few‐seeded bossiaea (*Bossiaea oligosperma*) can benefit an additional 106 threatened species, as reflected in the optimistic scenario (see Supporting Information). These results indicate the baseline scenario produced a credible and manageable list of umbrella priority species. However, we recognize that some species captured in our final baseline scenario list will not benefit from management, regardless of the level of management. These species include those that are no longer genetically robust (Frankel 1974), have population sizes that are too small (Daszak et al. 2000), are affected by uncertain or unmanageable threatening processes, have been subject to a sequence of unfortunate catastrophic events, or have populations that no longer occur across the full ecological gradient of their historical ranges (Crandall et al. 2000).

Another important consideration in prioritization strategies is species diversity (Levine & HilleRisLambers 2009). Managing species diversity ensures protection of a variety of habitats, ecological communities, and species (Arponen 2012; Polak et al. 2016). Our new umbrella method implicitly encourages diversity by forcing the algorithm to choose a variety of different areas. This forced variety is due to the decision rule that once a threat has been abated in 1 area that threat cannot be abated again. This is exemplified within our top 7 optimized species, whereby they were not only wide‐ranging (and hence fit into the umbrella notion), but also represented some of the major vegetation groups within Australia, including shorelines (i.e., Far Eastern Curlew), woodlands (i.e., koala), wetlands (i.e., Australasian Bittern), temperate forests (i.e., Regent Honeyeater), and tropical forests (i.e., Red Goshawk).

Although our method prioritizes conservation actions for umbrella species that maximize benefits to all other threatened flora and fauna, it should not be implemented in isolation of other planning efforts. There are many other considerations necessary for conservation success in Australia, including the protection of intact places (Watson et al. 2016), keeping species common (Simmonds et al. 2019; Wintle et al. 2019), proactive planning for climate change (Jones et al. 2016), and ensuring representation of all species and ecosystems (Pressey et al. 1993). Biodiversity conservation planning needs to be a strategic, coordinated, and interdisciplinary effort with a holistic conservation agenda.

As part of this holistic agenda, future researchers could extend this method by explicitly incorporating important conservation considerations, such as representation (Pressey et al. 1993) and area‐based targets (Tear et al. 2005). Representation and targets could be incorporated as estimates of what is adequate for biodiversity persistence (Rodrigues & Gaston 2001; Possingham et al. 2006). In addition to representation and targets, we recommend updating this prioritization effort once important information, such as the geographic distribution of threats and threat severity (Lawler et al. 2002) become available. In Australia, the location, extent, and severity of all threats affecting all threatened species are currently unknown; hence, our assumption of managing species for all threats across their full range. This future research is an important next step because this lack of knowledge may be contributing to the current failures in species recovery (Lawler et al. 2002).

As Earth experiences a biodiversity crisis with limited funding that constrains choice, governments need to utilize mathematically well‐formulated approaches that are efficient, objective, and transparent. Our problem‐based method for quantitatively choosing umbrella species that aims to maximize the total number of flora and fauna benefiting from management actions while remaining under a strict budget is such an approach. As we found, a clearly formulated prioritization approach that considers threats, actions, and costs can rationally choose umbrella species and result in the management of more biodiversity under a strict budget. We argue that nations around the world can markedly improve the selection of umbrella species with this prioritization approach.

## Supporting information

Additional description of methods (Appendix S1), results (Appendices S2, S3 S4, S5), costs (Appendix S5), and R code (Appendix S6) are available online. The authors are solely responsible for the content and functionality of these materials. Queries (other than absence of the material) should be directed to the corresponding author.
