# Supplementary material for: Use of surrogate species to cost‐effectively prioritize conservation actions
Source: Conserv Biol. 2019 Dec 26;34(3):600–10. doi: 10.1111/cobi.13430 (PMC7318674; doi:10.1111/cobi.13430)
Supplement: Supplementary file 1 — Additional description of methods (Appendix S1), results (Appendices S2, S3 S4, S5), costs (Appendix S5), and R code (Appendix S6) are available online. The authors are solely responsible for the content and functionality of these materials. Queries (other than absence of the material) should be directed to the corresponding author. [file COBI-34-600-s001.zip › cobi13430-sup-0001-SuppMat/cobi13430-sup-0001-Supp_PrioritySpecies_docR1.docx]

# **Supporting Information**

##### **METHODS**

**Species range intersections:**

Species range intersections were calculated using the PostGIS extension (version 2.3) within a PostgreSQL (version 10) database. All data were projected in a geodetic coordinate system (GDA94).

##### **Benefit Function: The Optimistic Scenario**

For the optimistic scenario, we assume that only one threat has to be managed to ensure persistence of a species under the umbrella, but we are willing to manage every threat impacting the priority umbrella species. An umbrella species can be any species that lives in the same space and is threatened by the same threats as other species (see figure S1).

##### **Benefit Function: The Pessimistic Scenario**

The pessimistic scenario assumes that all species must be managed for all threats throughout its entire geographic range (see Figure S1). This is the most conservative scenario, yet ensures that if there are interacting threats, adverse outcomes are kept to a minimum. For example, the presence of both cats (*Felis catus*) and foxes (*Vulpes vulpes*) has less of an impact on greater bilby (*Macrotis lagotis*), then just the presence of cats. Therefore, an adverse outcome may occur if only foxes are managed.


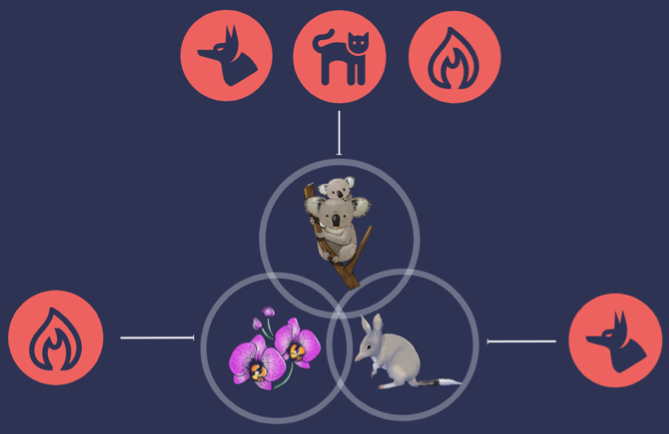


Figure S1. In the optimistic scenario, we manage fire, foxes (Vulpes vulpes), and cats (Felis catus) for koala (species i; Phascolarctos cinereus). It shares a common threat of foxes and overlaps greater bilby’s (Macrotis lagotis) distribution, and therefore receives a benefit of 1. Species i also shares a common threat of fire and overlaps orchid’s (Orchis) distribution, and therefore receives another benefit of 1. The greater bilby and the orchid will not be managed further and koala will receive a benefit of 3, including management of itself. In the intermediate case study, we assume that threats are partially managed based on their proportional overlap. In the intermediate scenario, the koala would receive a benefit of .25 (i.e. 25% of its distribution) for managing the action against foxes for greater bilby and .25 (i.e. 25% of its distribution) for managing the action against fire for orchid. Greater bilby and orchid will not be managed further than .75 and the koala will receive a benefit of 1.50. The pessimistic scenario assumes that all threats must be managed across the entire range of all species. In the above scenario, the koala, does not receive any benefit as no threat can be managed in its entirety.

##### **RESULTS**

Table S2. An overview of top 7 priority umbrella species under 3 different scenarios.

| Government List | | Optimistic List | | Baseline List | | Pessimistic List | |
| --- | --- | --- | --- | --- | --- | --- | --- |
| Species | Benefit | Species | Benefit | Species | Benefit | Species | Benefit |
| Botaurus_poiciloptilus | 15.04 | Bossiaea_oligosperma | 106.00 | Botaurus_poiciloptilus | 15.04 | Dianella_amoena | 2.17 |
| Anthochaera_phrygia | 6.85 | Thesium_australe | 17.00 | Phascolarctos_cinereus_(combined_populations_of_Qld,_NSW_and_the_ACT) | 9.99 | Cepobaculum_carronii | 1.50 |
| Numenius_madagascariensis | 5.31 | Brachyscias_verecundus | 8.00 | Anthochaera_phrygia | 6.85 | Acacia_axillaris | 1.50 |
| Myrmecobius_fasciatus | 2.98 | Epacris_exserta | 8.00 | Numenius_madagascariensis | 5.31 | Hipposideros_semoni | 1.50 |
| Dasyornis_brachypterus | 2.28 | Prasophyllum_pulchellum | 7.00 | Erythrotriorchis_radiatus | 4.55 | Caladenia_anthracina | 1.33 |
| Pseudocheirus_occidentalis | 1.34 | Epacris_virgata | 7.00 | Glycine_latrobeana | 4.02 | Macadamia_tetraphylla | 1.22 |
| Neophema_chrysogaster | 1.39 | Acacia_pharangites | 6.00 | Dianella_amoena | 3.20 | Arthraxon_hispidus | 1.22 |

Figure S3. Overview of threats impacting species.

Figure S4. Cost per unit decreases as the km2 area increases, representing economies of scale.

**R Code**

**S6. Baseline optimisation using Z value 0.30**

## Set up R

library(tidyverse)

threats_species_matrix <- read_csv("threats_species_matrix.csv")

Cost_threat_management <- read_csv("costs.csv")

species_int <- read_csv ("species_intersect.csv")

area <- read_csv("area.csv")

# Remove species duplicates in the same row

species_int <- subset(species_int, sp_a != sp_b)

# Obtain new overlaps due to duplicates

species_int <- species_int %>% group_by(sp_a,sp_b) %>% summarise(

n=n(),

b_area = sum(unique(KM2_sp_b)),

isect_area = sum(unique(Overlap_km)),

prop_overlap = isect_area/b_area

)

# Plotting the diminishing returns

area_cost_scaling_factor <- 0.3

# Calculating all species benefits, considered individually

U <- matrix(1,nrow=nrow(threats_species_matrix),ncol=nrow(Cost_threat_management))

species_benefits <- function(){

rs <- data.frame(species_id = threats_species_matrix$species_id, B = NA, C = NA)

for (i in 1:nrow(rs)){

overlaps <- subset(species_int,sp_a == threats_species_matrix$species_id[i])

Tik = as.numeric(threats_species_matrix[i,5:46]) # this is a vector since we consider only one i at a time

Uik = U[i,]

if (nrow(overlaps)==0){

cat("No overlaps found for species ", i, ", ",threats_species_matrix$species_id[i], "\n")

# cat("Setting benefit to zero \n")

rs[i,"B"] = threats_species_matrix$weight[i] * sum(Tik * Uik) / sum(Tik)

rs[i,"C"] = sum(Tik * Cost_threat_management$Cost_km_year) * subset(area,ALL_MAPP_2==threats_species_matrix$species_id[i])$KM2[1]^area_cost_scaling_factor

next

}

mtch <- match(overlaps$sp_b,threats_species_matrix$species_id)

overlaps$W <- threats_species_matrix$weight[mtch]

Tjk = as.matrix(threats_species_matrix[mtch,5:46])

overlaps$total_threats <- apply(Tjk,1,sum)

Ujk = U[mtch,]

overlaps$common_threats <- as.numeric((Ujk*Tjk) %*% Tik)

overlaps$benefit_to_j <- with(overlaps, prop_overlap * W * common_threats / total_threats)

overlaps <- subset(overlaps,!is.na(W) & total_threats>0)

rs[i,"B"] = sum(overlaps$benefit_to_j)

rs[i,"C"] = sum(Tik * Uik * Cost_threat_management$Cost_km_year) * subset(area,ALL_MAPP_2==threats_species_matrix$species_id[i])$KM2[1]^area_cost_scaling_factor

rs[i,"B"] = rs[i,"B"] + threats_species_matrix$weight[i] * sum(Tik * Uik) / sum(Tik) ## to stop double counting benefit of self

}

rs$E = rs$B/rs$C

return(rs)

}

# Run code

benefits <- species_benefits()

write_csv(benefits, "benefits_baseline_0.30_lower.csv")

# adjusting U

adjust_U <- function(U,species){

i = which(threats_species_matrix$species_id == species)

U[i,] <- 0

overlaps <- subset(species_int, sp_a == threats_species_matrix$species_id[i])

mtch <- match(overlaps$sp_b,threats_species_matrix$species_id)

overlaps <- overlaps[!is.na(mtch),]

mtch <- mtch[!is.na(mtch)]

Tik = as.numeric(threats_species_matrix[i,5:46])

U[mtch,] <- U[mtch,] * (1-outer(overlaps$prop_overlap,Tik,"*"))

return(U)

}

# Remove top species

U <- adjust_U(U,"Bossiaea_oligosperma")

benefits2 <- species_benefits()
